# Evolutionary divergence of motifs in B-class MADS-box proteins of seed plants

**DOI:** 10.1186/s40709-021-00144-7

**Published:** 2021-05-28

**Authors:** Gangxu Shen, Yong Jia, Wei-Lung Wang

**Affiliations:** 1 School of Chinese Medicine for Post-Baccalaureate, I-Shou University, Kaohsiung, 84001 Taiwan; 2 Department of Biology, National Changhua University of Education, Changhua, 500 Taiwan; 3 College of Science, Health, Engineering and Education, Murdoch University, Murdoch, WA 6150 Australia

**Keywords:** B gene, MADS-box gene, MADS domain, MEME, *Amborella trichopoda*

## Abstract

**Background:**

MADS-box transcription factors function as homo- or heterodimers and regulate many aspects of plant development; moreover, MADS-box genes have undergone extensive duplication and divergence. For example, the morphological diversity of floral organs is closely related to the functional divergence of the MADS-box gene family. B-class genes (such as *Arabidopsis thaliana APETALA3* [*AP3*] and *PISTILLATA* [*PI*]) belong to a subgroup of MADS-box genes. Here, we collected 97 MADS-box B protein sequences from 21 seed plant species and examined their motifs to better understand the functional evolution of B proteins.

**Results:**

We used the MEME tool to identify conserved sequence motifs in these B proteins; unique motif arrangements and sequences were identified in these B proteins. The keratin-like domains of *Malus domestica* and *Populus trichocarpa* B proteins differed from those in other angiosperms, suggesting that a novel regulatory network might have evolved in these species. The MADS domains of *Nelumbo nucifera*, *Glycine max*, and *Amborella trichopoda* B-proteins contained motif 9; in contrast, those of other plants contained motif 1. Protein modelling analyses revealed that MADS domains with motif 9 may lack amino acid sites required for DNA-binding. These results suggested that the three species might share an alternative mechanism controlling floral development.

**Conclusions:**

*Amborella trichopoda* has B proteins with either motif 1 or motif 9 MADS domains, suggesting that these two types of MADS domains evolved from the ancestral domain into two groups, those with motif 9 (*N. nucifera* and *G. max*), and those with motif 1. Moreover, our results suggest that the homodimer/heterodimer intermediate transition structure first appeared in *A. trichopoda*. Therefore, our systematic analysis of the motifs in B proteins sheds light on the evolution of these important transcription factors.

**Supplementary Information:**

The online version contains supplementary material available at 10.1186/s40709-021-00144-7.

## Background

The versatile MADS-box transcription factors (TFs) shape development in most multicellular eukaryotes; however, terrestrial plants have more MADS-box genes than other eukaryotic groups [1–4]. Indeed, during the evolution of flowering plants, MADS-box genes had key roles in shaping the diverse structures of flowers [5, 6] and these diverse structures (as well as seed-based propagation) in turn had key roles in the success of flowering plants [7]. Therefore, studies of MADS-box genes provide insight into the development of the diverse flower types found in angiosperms [8]. The term MADS-box gene is derived from four of the earliest recognized family members: *MCM1*, *AGAMOUS*, *DEFICIENS*, and *serum response factor* (*SRF*), from *Saccharomyces cerevisiae*, *AGAMOUS* from *Arabidopsis thaliana*, *DEFICIENS* from *Antirrhinum majus*, and *serum response factor* (*SRF*) from *Homo sapiens* [8, 9], respectively. During evolution, MADS-box genes underwent multiple duplications and divergence, producing two major clades: the Serum Response Factor-like clade (type I) and the Myocyte Enhancer Factor-2-like clade (type II) [10, 11]. Charophyte algae and terrestrial plants have MIKC-type TFs that contain MADS, Intervening, Keratin-like, and C-terminal (MIKC) domains [12, 13]. MIKC-type TFs can be further divided into the MIKC* and the MIKC^C^ groups [14].

One subgroup of the MIKC^C^-type MADS-box genes, the B genes, are defined from *Arabidopsis thaliana APETALA3* (*AP3*) and *PISTILLATA* (*PI*), which function in the specification of floral organs. B genes are not present in bryophytes and seedless vascular plants [15, 16]. Our previous research [17] confirmed the evolutionary history of the ABCDE and AGL6 genes and clarifies their evolutionary path. Here, to shed light on the evolution of B genes, we examined B proteins from gymnosperm, basal angiosperm, monocot, and magnoliopsida/eudicot species, using 97 B protein sequences from 21 seed plant species (Table 1) to identify conserved motifs.

**Table 1:** 97 *AP3/PI* query sequences in 21 seed plants

No	Species	*AP3/PI*	No	Species	*AP3/PI*	No	Species	*AP3/PI*
1	*Gnetum gnemon*	*GGM2*	11	*Carica papaya*	*CpMADS22*	16	Populus trichocarpa*	*PtMADS10*
2	*Ginkgo biloba*	*GbMADS4*			*CpMADS23*			*PtMADS11*
		*GbMADS9*			*CpMADS24*			*PtMADS22*
3	***Amborella trichopoda***	***LOC18424280***	12	Malus domestica*	*MdMADS13P*			*PtMADS25*
		*LOC18448591*			*MdMADS31*			PtMADS30*
		***LOC18429933***			*MdMADS64*			PtMADS38*
		*LOC18436882*			MdMADS65*			PtMADS45*
4	*Oryza sativa*	*OsMADS2*			MdMADS99*	17	*Linum usitatissimum*	*Lu MADS34*
		*OsMADS4*			*MdMADS105*			*LuMADS67*
		*OsMADS16*			MdMADS121*			*LuMADS69*
5	*Zea mays*	*ZmMADS20*			*MdMADS124*			*LuMADS74*
		*ZmMADS60*			MdMADS127*			*LuMADS94*
6	*Phalaenopsis aphrodite*	*PATC133864*			*MdMADS131*			*LuMADS117*
		*PATC138350*			*MdMADS134*	18	*Solanum tuberosum*	*LuMADS120*
		*PATC152852*			*MdMADS139*			*StMADS54*
		*PATC154853*			MdMADS151*			*StMADS61*
		*PATC240636*	13	*Cucumis sativus*	*CsMADS21*			*StMADS86*
7	*Musa acuminata*	*MaMADS6p*			*CsMADS23*			*StMADS112*
		*MaMADS14*	14	***Glycine max***	*GmMADS4*			*StMADS145*
		*MaMADS71*			*GmMADS5*			*StMADS153*
		*MaMADS88*			*GmMADS6*			*StMADS253*
8	*Arabidopsis thaliana*	*AT3G54340*			*GmMADS7*	19	*Ricinus communis*	*RcMADS30*
		*AT5G20240*			*GmMADS8*			*RcMADS33*
9	*Vitis vinifera*	*VvAP3_1*			*GmMADS9*			*RcMADS34*
		*VvAP3_2*			*GmMADS10*	20	*Manihot esculenta*	*MeMADS45*
		*VvPI*			*GmMADS110*			*MeMADS46*
10	*Citrus sinensis*	*CsiMADS33*			***GmMADS121***			*MeMADS47*
		*CsiMADS34*			***GmMADS133***			*MeMADS48*
		*CsiMADS35*			***GmMADS147***			*MeMADS52*
		*CsiMADS41*			***GmMADS175***	21	*Solanum lycopersicum*	*TM6*
		*CsiMADS42*	*15*	***Nelumbo nucifera***	***ABE11602***			
		*CsiMADS43*			***ADD25193***			
		*CsiMADS46*			***ADD25194***			
					***ADD25195***			

The name of the bolded species are significantly different in M domain. The name of the asterisks* species are significantly different in K1 domain. Gymnosperms (number 1–2) and angiosperms (number 3–21): Basal angiosperm (3. *Amborella trichopoda*), Monocots (4. *Oryza sativa*, 5. *Zea mays*, 6. *Phalaenopsis aphrodite*, and 7. *Musa acuminata*), and magnoliopsida and eudicots (8–21)

## Results

### Identification of B class gene sequences

We retrieved 97 known B protein sequences (Table 1) using proteins from *A. thaliana* (*AT3G54340* and *AT5G20240*) and *Oryza sativa* (*OsMADS2, 4,* and *16*) as query sequences [1, 3, 6, 18–20] in a Basic Local Alignment Search Tool (BLAST) search [20]. Subsequently, the retrieved sequences were entered into Simple Modular Architecture Research Tool (SMART) to confirm they have MADS-box domains [22]. Sequence alignment of the MADS domains was displayed in Additional file 2: Fig. S1.

### Motif identification in B class genes

**Fig. 1:**
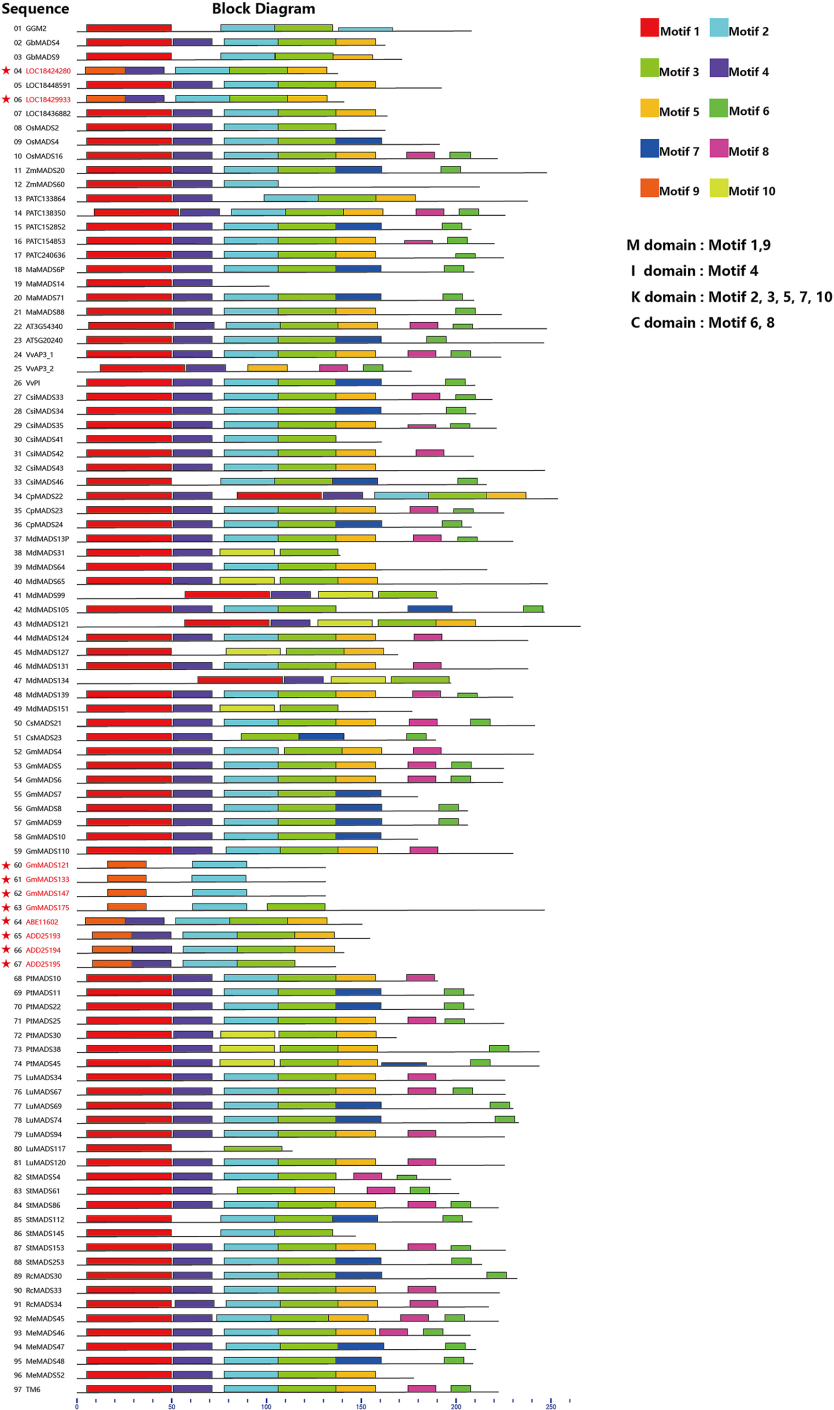
MEME search results regarding the protein motifs in AP3/PI. The protein motifs of the B proteins in 97 sequences were separately obtained using the MEME motif search tool for each group. Ten motifs were identified, each of which is represented as a colored box. Asterisks and red letters: motif 9. Gymnosperms (numbers 1–3) and angiosperms (numbers 4–97): Basal angiosperm (*Amborella trichopoda*: 4–7), monocots (*Oryza sativa*: 8–10, *Zea mays*: 11–12, *Phalaenopsis aphrodite*: 13–17 and *Musa acuminata*: 18–21), and magnoliopsida and eudicots (22–97)

We used the MEME tool [3] to identify conserved sequence motifs in 97 B protein sequences in this study (Fig. 1 and Additional file 1). A total of 10 conserved motifs were identified in the AP3/PI proteins among 21 plant species (Fig. 1). The detailed amino acid sites conservation profiles are shown in Additional file 2: Fig. S2. Motif 1 (consensus sequence IEIKRIENPTNRQVTYSKRRNGIFKKAHELTVLCDAKVSLIMFSS) and motif 9 (consensus sequence KAAELTVLCDAKVSLIMFSST) overlap with the MADS domain (M domain). In the B proteins of *Nelumbo nucifera* (*ABE11602*, *ADD25193*, *ADD25194*, and *ADD25195*), *Glycine max* (*GmMADS121*, GmMADS133, *GmMADS147*, and *GmMADS175*), and some of the B proteins in *Amborella trichopoda* (*LOC18429933* and *LOC18424280*), motif 1 was replaced by motif 9 (Fig. 1). However, as shown in Fig. 1, some *A. trichopoda* B protein sequences have motif 1 (*LOC18436882*, and *LOC18448591*). The M domains of gymnosperm B proteins belong to the motif 1 group (Fig. 1).

Gymnosperm B proteins (*GbMADS4, GbMADS9*, and *GGM2*) lack motif 4, which overlaps with the intervening domain (I domain), consists of approximately 30 amino acids and is a less-conserved region involved in protein dimerization [10].

Motifs 2, 3, 5, 7, and 10 were found in the keratin-like domain (K-domain). The K domain of AP3/PI consists of approximately 70 amino acids that are divided into K1, K2, and K3 subdomains [13]. K1 and K2 are required for dimer formation, whereas K3 may participate in multimerization [13, 23]. We found motif 2 and motif 10 in the K1 subdomain; motif 3 was found in K2; and motif 5, with the consensus sequence KYHVIKTQTDTCKKKVRNLEE, or its alternative motif 7, with the consensus sequence QMEYWKMMKRNDKMLEDENKQLTF, were found in K3 (Figs. 1 and Additional file 2: Fig. S2).

Motif 10 of *Malus domestica* (*MdMADS65, MdMADS99, MdMADS121, MdMADS127*, and *MdMADS151*) and *Populus trichocarpa* (*PtMADS30, PtMADS38*, and *PtMADS45*) replaced motif 2 in the K1 domain of other angiosperms. Motifs 6 and 8 were found in the C-terminal domain (C domain) (Fig. 1).

### Phylogeny analyses of plant *AP3/PI* genes

**Fig. 2:**
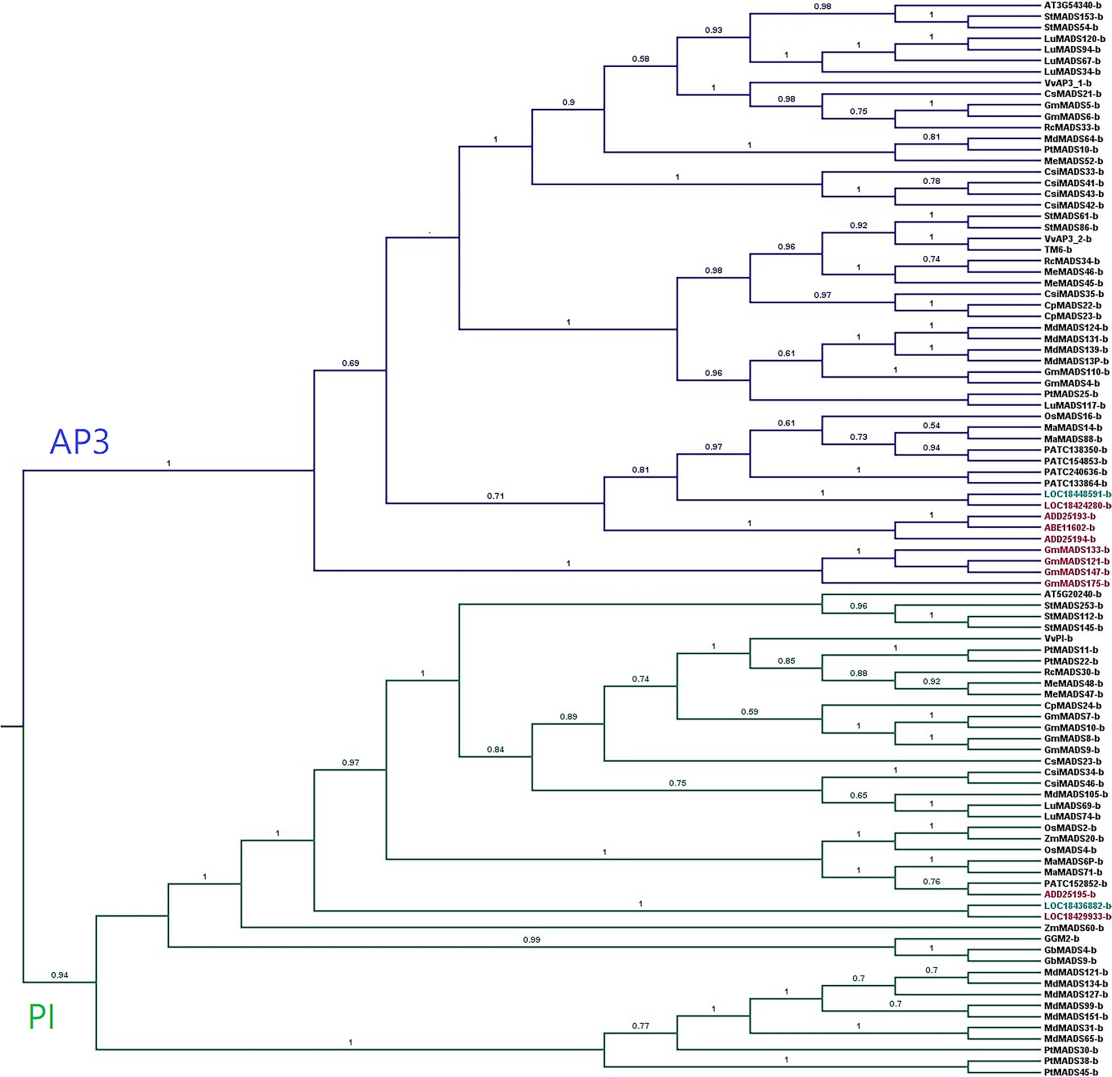
Phylogenetic tree of the plant AP3/PI genes. The phylogeny was reconstructed using Bayesian approach. Bayesian posterior probability was annotated above each branch in the phylogenetic tree. M domain: motif 1(black and green; green: *A. trichopoda*: *LOC18448591* and *LOC18436882*) and motif 9 (red): *LOC18424280, LOC18429933, GmMADS121, GmMADS133, GmMADS147, GmMADS175, ABE11602, ADD25193, ADD25194*, and *ADD25195*

To investigate the phylogenetic relationship among these 97 *AP3/PI* sequences (Additional file 1), a Bayesian phylogeny was reconstructed (Fig. 2). Overall, the determined phylogeny was consistent with the species tree, indicating the phylogeny was reliable. As shown in Fig. 2, the target genes have divided into two major groups, representing *AP3* and *PI*, respectively. Those MADS protein sequences containing motif 9 (highlighted in red) were identified within both *AP3* group (*LOC18424280, ABE11602, ADD25194, GmMADS121, GmMADS133, GmMADS147*, and *GmMADS175*) and the *PI* group (*ADD25195* and *LOC18429933*), indicating motif 9 may have evolved independently within the two separate lineages. The position of *Amborella* MADS genes (gene IDs starts with LOC) was consistent with this species as an evolutionary intermediate between lower plants and core eudicot plants. Noteworthy, *Amborella* MADS genes *LOC18448591* and *LOC18436882* (highlighted in green) containing motif 1 were identified both in *AP3* and *PI* groups (Fig. 2).

### Protein structural modelling of MADS domain

**Fig. 3:**
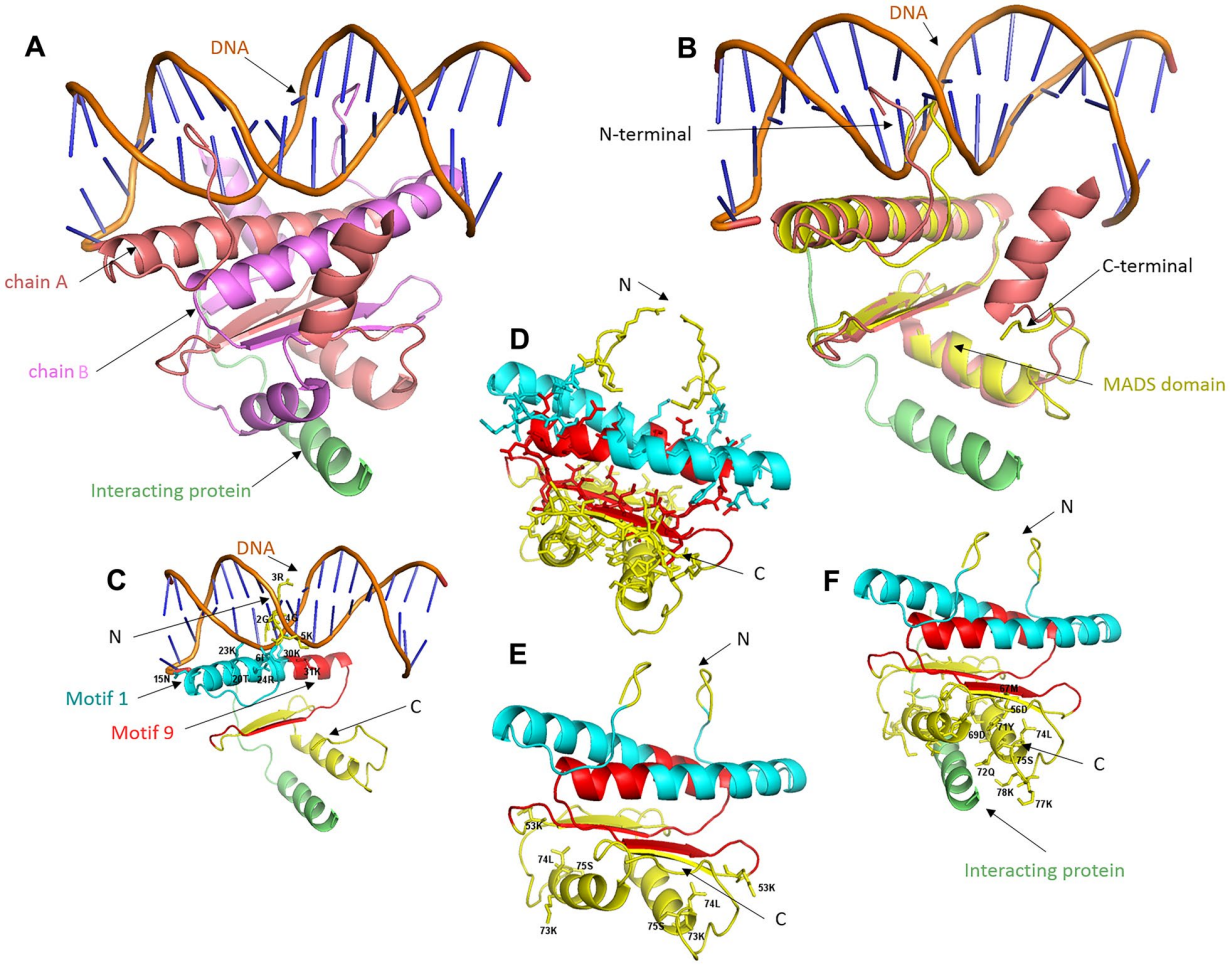
Protein structural modelling of plant MADS domain (*AT5G20240*). **A** Overall structure (PDB: 1TQE) of human MEF2 (dimers) in complex with DNA and interacting protein (green color). **B** Superimposition of modelled *AT5G20240* (yellow) with MEF2 (deep salmon). **C** Displays the spatial locations of motif 1 (cyan & red) and motif 9 (red), with DNA-binding residues shown in sticks. **D** Displays the identified dimerization residues in sticks. **E** Displays potential tetramerization residues in sticks. **F** Displays residues (sticks) involved in protein–protein interaction. Amino acid numbering is according to the M domain of *AT5G20240*

To investigate the potential impact of the substitution between motif 1 and motif 9, protein structural modelling was performed for plant MADS domains using *AT5G20240* as a reference. The previously well-determined structure of human myocyte enhancer factor-2 (MEF2, PDB ID: 1TQE, MADS-box superfamily) in complex with DNA and its interacting protein (Fig. 3A) was used as a template for homology (~ 48% aa identity) structural modelling. Structural superimposition (Fig. 3B) showed the MADS domain of *AT5G20240* was well-conserved in comparison with MEF2. Based on 3D structural superimposition with MEF2, the spatial positions of motif 1 and motif 9 of *AT5G20240* in reference to binding DNA were displayed (Fig. 3C). A total of 10 amino acids in *AT5G20240* were identified as potential DNA-binding sites (shown in sticks in Fig. 3C, Additional file 2: Fig. S3), six of which were located in motif 1 versus only one in motif 9, suggesting motif 1, instead of motif 9, may be responsible for DNA-binding.

As indicated in the MEF2 structure, the MADS domain binds DNA in the dimer form. Thus, amino acid sites for dimerization were also identified for AT5G20240 (Fig. 3D). A total of 52 residues out of the 89 MADS domain amino acids were identified for dimerization interaction (Fig. 3D, Additional file 2: Fig. S3), which cover the majority of motif 9 residues and also a significant number of residues in motif 1. Motif 1, which overlaps with motif 9, was intertwined for the MADS dimers to form a dimer complex (Fig. 3D & E), suggesting that both motif 1 and motif 9 were critical for the dimerization interaction. In addition, residues for protein–protein interaction (Fig. 3E) and potential tetramerization (Fig. 3F) were also identified and displayed. None of these interactions involved residues from motif 1 and motif 9.

## Discussion

The MADS domains in *N. nucifera*, *G. max*, and *A. trichopoda* contain motif 9, whereas the MADS (M) domain of the B proteins from other plants contain motif 1. The two motifs overlap; motif 9 is a shorter version of motif 1, missing the first half. It has been suggested that all MADS TFs function as biological dimers [24, 25]. Among the MIKC domains, residues in domains M, I, and K are involved in the dimerization process [25, 26]. In this study, protein structural modelling suggested motif 1 and motif 9 in the M domain were mainly involved in the dimerization process. Based on the determined structure of human MEF2 [26], it seems that the formation of a dimer is critical for the DNA-binding process. For example, our modelling results showed that the third residue arginine in the M domain was responsible for both DNA binding and dimerization. Indeed, a previous study has proven that this residue is critical for the recognition of DNA binding sites [27]. Notably, the third arginine residue and several other residues identified for DNA binding in this study were missing in motif 9 compared to a complete motif 1. Therefore, the variation between motif 1 and motif 9 may affect the formation of functional MADS proteins. These observations suggested that either MADS proteins with motif 9 identified in *N. nucifera*, *G. max*, and *A. trichopoda* are dysfunctional or that these three species may share an alternative control mechanism for floral development. Moreover, differences in gene expression support the potential divergence of floral development in these species. In our study, three *N. nucifera* genes (*ABE11602*[*Nenu.AP3*], *ADD25193* [*Nenu.AP3-1*], and ADD25194 [*Nenu.AP3-2*]) were placed in the *AP3* and *ADD25195* belongs to *PI* (Fig. 1). Indeed, three *N. nucifera PI* and *AP3* homologs (*Nenu.PI, Nenu.AP3-1,* and *Nenu.AP3-2*) are expressed in petals and stamens, similar to other B genes in eudicots [28]. *Nenu.PI* is additionally expressed in sepals, while *Nenu.AP3-1* is also detected in carpels. [28]. Therefore, sepal and petal differentiation in *N. nucifera* might be related to the alternative M domains in its B genes.

The observation that some *A. trichopoda* B protein sequences contain motif 9 and others have motif 1 suggests that the M domains of angiosperm B proteins evolved from an ancestor of *A. trichopoda* into two groups: a few species have B proteins with M domains that have motif 9 (*N. nucifera* and *G. max*) and most have motif 1. The M domains of gymnosperms belong to the motif 1 group (Fig. 1), so we speculate that the motif-1 M domain evolved earlier than motif 9. The B protein repertoire of *A. trichopoda* has both types of M domains, potentially increasing its capacity for floral organ diversity.

Studies of B proteins from across plant species have shed light on the evolution of their protein–protein interactions. For example, a gymnosperm B protein from *Gnetum gnemon* (motif 1) binds DNA as a homodimer [29]. Our protein modelling analyses also supported that plant MADS proteins function as a biological dimer, in which the dimerization is critical for target DNA-binding. The monocot *Lilium regale* has two classes of B proteins: the GLOBOSA (GLO)-like proteins (such as PI) homodimerize, but the DEFICIENS-like proteins (such as AP3) form heterodimers with GLO-like proteins [29]. These data suggest that B proteins evolved to form homodimers and then to form heterodimers, but in the evolution of these two structures, homodimer/heterodimer transition structures appeared. The MADS domain is important for DNA binding and dimerization [29]. As shown in Fig. 1, *A. trichopoda* has a complex repertoire of M domains (motifs 1 and 9). Based on structural modelling, this study identified the specific amino acid residues responsible for dimerization interactions, which involved a significant number of residues located in motif 1 and motif 9. Analysis of conserved motifs in B proteins reveals that the homodimer/heterodimer intermediate transition structure may have first appeared in the ancestors of *A. trichopoda* (Fig. 2), because *A. trichopoda* genes *LOC18448591* and *LOC18436882* (highlighted in green, Fig. 2) containing motif 1, as well as *LOC18424280* and *LOC18429933* (highlighted in red, Fig. 2) containing motif 9, were identified both in *AP3* and *PI* groups.

Many of the other motifs identified here affect domains involved in protein–protein interactions. For example, motif 4 is in the I domain, which is involved in protein dimerization [10]; it was surprising to find that motif 4 was absent in gymnosperm B proteins. Motifs 2, 3, 5, 7, and 10 were found in the K domain and the K1 and K2 subdomains are required for dimer formation, whereas K3 may participate in multimerization [13, 23]. Moreover, motif 10 of *Malus domestica* and *Populus trichocarpa* B proteins replaced motif 2 in the K1 domain of other angiosperms, suggesting that an alternative regulatory network might have evolved in these two species. Shan et al. have put forward similar views in a previous paper [30], but in this study, the related protein motif analysis was performed after *A. trichopoda* was sequenced [31]. Therefore, our research results can objectively confirm this result. Molecular evolutionary analyses provide a powerful approach for the identification of changes in amino acids, possibly associated with the evolution of gene function [30]. It is hypothesized that homodimerization or heterodimerization of the AP1/SQUA, AP3/PI, AG, and SEP-like proteins function as master regulators of the floral development in *Arabidopsis thaliana* [32].

## Conclusions

According to our protein motif analysis, the B proteins of seed plants exhibited unique motif arrangements and sequences that might affect their floral development. For example, the differentiation of sepals and petals in *N. nucifera* might be related to the alternative M domains in their B proteins. *Amborella trichopoda* has a complex repertoire of M domains and this may affect its floral organ diversity. Moreover, our results shed light on B protein evolution, suggesting that the M domains of angiosperms evolved from an ancestor of *A. trichopoda* to form two groups: a small group with the motif 9 M domain (*N. nucifera* and *G. max*), and most angiosperms with the motif 1 M domain. Due to the M domains of gymnosperm B proteins, belonging to the motif 1 group, we speculated that the evolution of the motif 1 group occurred earlier than that of the motif 9 group. Finally, our results provide insight into the evolution of protein–protein interactions, suggesting that interaction and dimerization critically rely on both motif 1 and motif 9 groups. The homodimer/heterodimer intermediate transition structure may have first appeared in *A. trichopoda*, because of the fact that the *A. trichopoda* motif 1 (*LOC1844859*1 and *LOC18436882*) and motif 9 (*LOC18424280* and *LOC18429933*) were both identified in *AP3* and *PI* groups. Moreover, the B proteins (K1 domain) of *M. domestica* and *P. trichocarpa* differ from those in other angiosperms, suggesting that an alternative regulatory network might have evolved in these two species. Our results will assist researchers in exploring MADS-box protein functional evolution.

## Methods

### Species selection

The selection of representative gymnosperm species was performed from a range of families, including Gnetaceae (*G. gnemon*), Pinaceae (*Picea abies*), Podocarpaceae (*Podocarpus macrophyllus*), Araucariaceae (*Wollemia nobilis*), Sciadopityaceae (*Sciadopitys verticillata*), Taxaceae (*Taxus baccata*), Cupressaceae (*Cryptomeria japonica*), and Ginkgoaceae (*G. biloba*); however, but we found that only *G. gnemon* and *G. biloba* were found to have the B gene [17]. When selecting angiosperms, we included species from the three groups: (1) basal angiosperms (*A. trichopoda*), (2) monocots (*Osativa*, *Zea mays*, *Phalaenopsis aphrodite*, and *Musa acuminata*), and (3) magnoliopsida and eudicots. Since magnoliopsida and eudicots are the largest group of angiosperms, we chose to include 14 typical species from the different families in this group. We considered choosing these seed plants (gymnosperms and angiosperms) to represent a complete evolution of plants, which is of crucial importance for the phylogenetic analysis. Furthermore, MADS-box genes are present in bryophytes and seedless vascular plants, whereas ABCDE and AGL6 genes have MADS-box genes but do not have ABCDE or AGL6 [33].

### Sequence retrieval

Query sequences were obtained from the following databases: *A. thaliana*, http://www.arabidopsis.org/; *O. sativa*, http://rice.plantbiology.msu.edu/; *P. aphrodite*, http://orchidstra.abrc.sinica.edu.tw; Gramene, http://www.gramene.org/; National Center for Biotechnology Information (NCBI), http://www.ncbi.nlm.nih.gov/; Phytozome, http://www.phytozome.net/; and UNIPROT, http://www.uniprot.org/uniprot/. B protein sequences from *A. thaliana* and *O. sativa* were used for BLAST-P queries against available genomic data.

### MADS-box sequences validation

Online tool SMART (http://smart.embl-heidelberg.de/) was used to validate the presence of the MADS domains in the proteins encoded by the target genes [22].

### Conserved motif prediction using MEME

MEME [3] online tool (meme.sdsc.edu/meme/meme-intro.html) was used to identify conserved motifs. A MEME search was performed with the following settings: (1) optimum motif width was set to ≥ 6 and ≤ 50; (2) the maximum number of motifs identified was set to 10 and (3) occurrences of a single motif were distributed among the sequences with the setting “zero” or “one per sequence” (-mod zoops).

### Sequence alignment and phylogeny reconstruction

The amino acid sequences were aligned using the program MUSCLE version 3.8.31 [34] with 8 iterations. The phylogeny was searched by Bayesian simulation implemented using BEAST2 [35] program. The strict molecular clock assumption with an unlinked substitution model Yule + G (five categories) was used. A single Markov Chain Monte Carlo chain was run for 1,000,000 generations (sampled every 1000) with the first 1000 trees as pre burn-in until convergence. The final phylogenetic tree was inferred by treeannotator [35], with the first 5% trees discarded.

### Protein structural modelling

Homology structural modelling of plant MADS domain was performed using Modeller [25] server based on sequence alignment in Chimera. Human MEF2 (PDB: 1TQE) [36] in complex with binding DNA and interacting protein was selected as the template (48% aa identity). The quality of the generated model was assessed based on lowest discrete optimized protein energy (DOPE) values and GA 341 score of 1, which indicated the reliability of these models. Potential interacting residues were identified based on distance less than 4 Å using PyMol 1.3r1 (Schrodinger, LLC; https://pymol.org). All structural visualizations were performed using PyMol.

## Supplementary Information

**Additional file 1.** 97 B protein sequences.

**Additional file 2**: **Fig. S1**. Sequence alignment of MADS domains of plant AP3/PI genes ordered by the developed phylogeny. Motif 9 were highlighted in red box. **Fig. S2**. Conserved sequence profiles for motif 1-10. **Fig. S3**. Identified interacting residues in MADS domain modelling. A) DNA-binding residues. B) Dimerization residues. C) Protein-protein interaction residues. D) Potential tetramerization residues. Cyan & red color indicate motif 1. Red color indicates motif 9. The other residues are in yellow color. The identified residues are highlighted in each row.

## Data Availability

The *O. sativa* database (http://rice.plantbiology.msu.edu/), the *A. thaliana* database (http://www.arabidopsis.org/), NCBI (http://www.ncbi.nlm.nih.gov/), Gramene (http://www.gramene.org/), UNIPROT (http://www.uniprot.org/uniprot/), Phytozome (http://www.phytozome.net/), and *P. aphrodite* database (http://orchidstra.abrc.sinica.edu.tw).

## Abbreviations

AGL6: AGAMOUS- LIKE 6
AP: APETALA
BEAST: Bayesian evolutionary analysis by sampling trees
BLAST: Basic Local Alignment Search Tool
DOPE: Discrete optimized protein energy
PI: PISTILLATA
SMART: Simple Modular Architecture Research Tool
SRF: Serum response factor
