# Supplementary material for: Evolutionary divergence of motifs in B-class MADS-box proteins of seed plants
Source: J Biol Res (Thessalon). 2021 May 28;28:12. doi: 10.1186/s40709-021-00144-7 (PMC8161959; doi:10.1186/s40709-021-00144-7)

**Evolutionary Divergence of Motifs in B-class MADS-box Proteins of Seed Plants**

Gangxu Shen^1,2,*,†^, Yong Jia^3,†^,Wei-Lung Wang^2,*^

^1^ School of Chinese Medicine for Post-Baccalaureate, I-Shou University, Kaohsiung 84001, Taiwan

^2^ Department of Biology, National Changhua University of Education, Changhua 500, Taiwan

^3^ College of Science, Health, Engineering and Education, Murdoch University, Murdoch, WA, 6150, Australia

†These authors have contributed equally to the study

Yong Jia: y.jia@murdoch.edu.au

*Correspondence:

Gangxu Shen:numbershen@yahoo.com.tw

Wei-Lung Wang: wlwang@cc.ncue.edu.tw

**Supplementary files** **Fig. S1-S3**

**Fig. S1. Sequence alignment of MADS domains of plant AP3/PI genes ordered by the developed phylogeny**. Motif 9 were highlighted in red box.


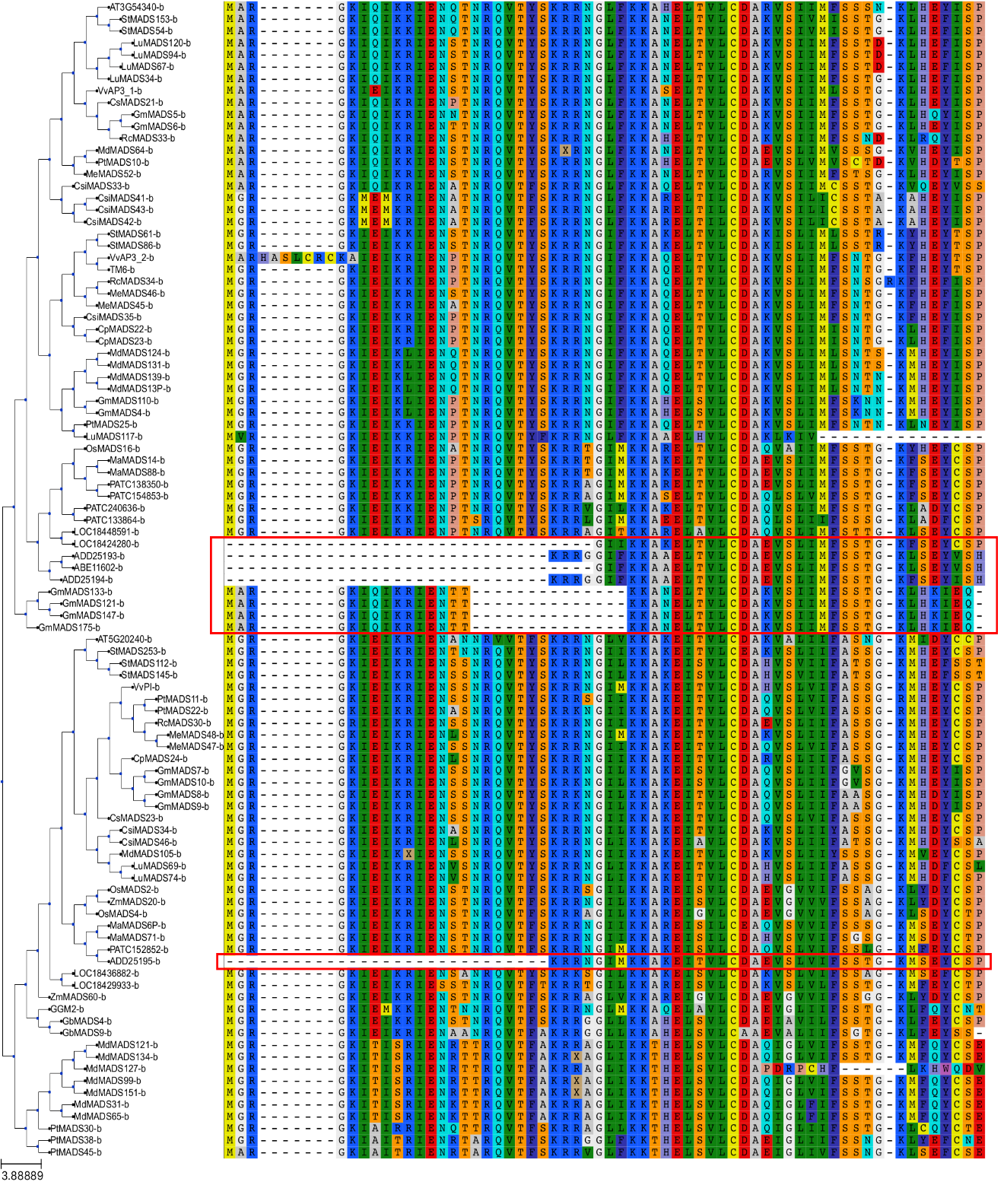


**Fig. S2. Conserved sequence profiles for motif 1-10**.


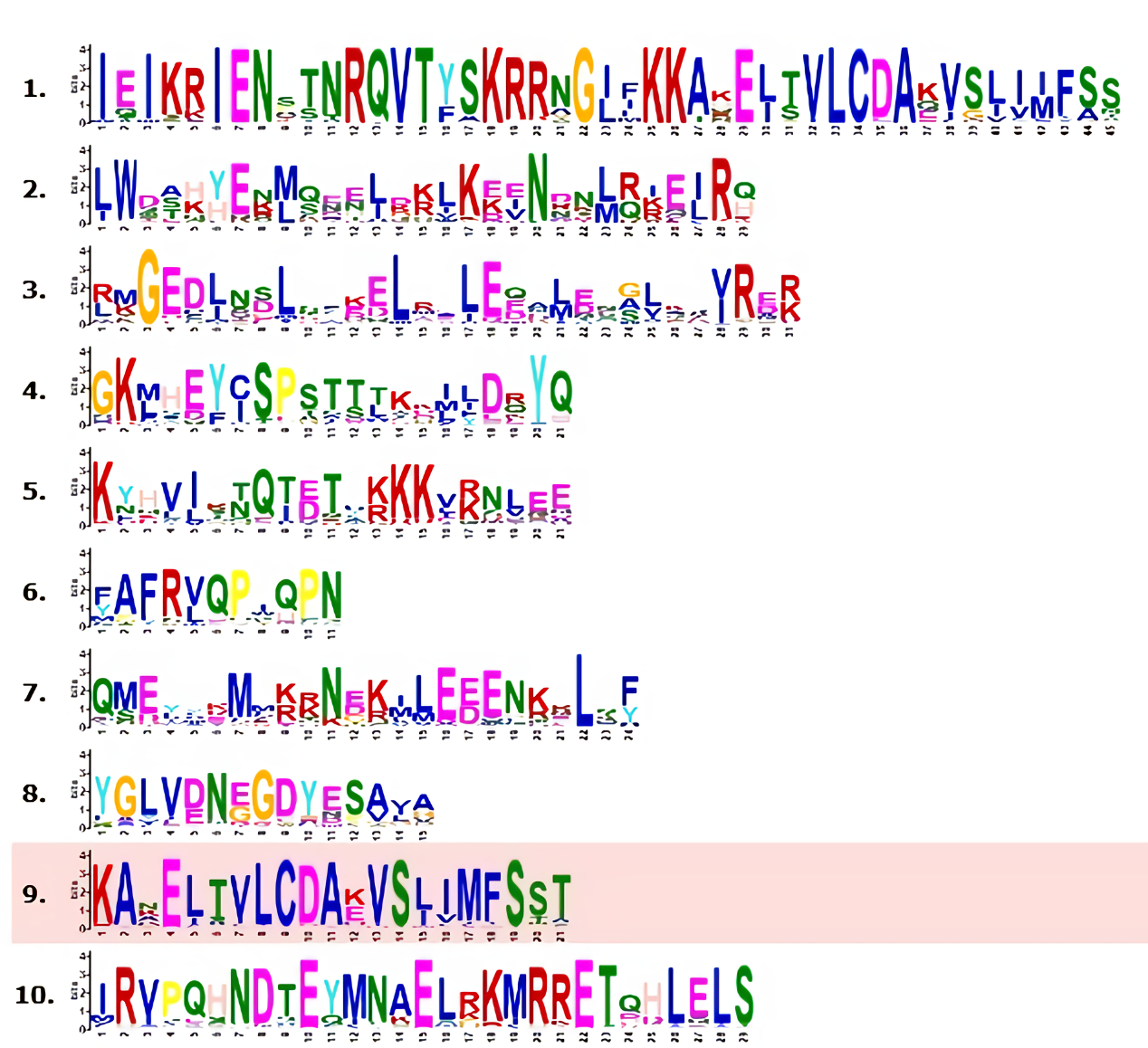


**Fig. S3. Identified interacting residues in MADS domain modelling**. **A**) DNA-binding residues. **B**) Dimerizaiton residues. **C**) Protein-protein interaction residues. **D**) Potential tetramerization residues. Cyan & red color indicate motif 1. Red color indicates motif 9. The other residues are in yellow color. The identified residues are highlighted in each row.


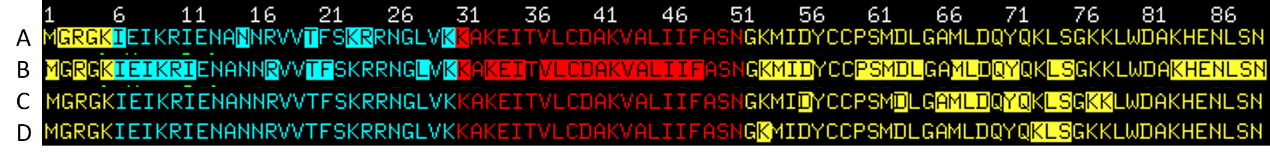

Supplement: Supplementary file 2 — Additional file 2: Fig. S1. Sequence alignment of MADS domains of plant AP3/PI genes ordered by the developed phylogeny. Motif 9 were highlighted in red box. Fig. S2. Conserved sequence profiles for motif 1-10. Fig. S3. Identified interacting residues in MADS domain modelling. A) DNA-binding residues. B) Dimerization residues. C) Protein-protein interaction residues. D) Potential tetramerization residues. Cyan & red color indicate motif 1. Red color indicates motif 9. The other residues are in yellow color. The identified residues are highlighted in each row. [file 40709_2021_144_MOESM2_ESM.docx]
